# The Impact of Ground Tire Rubber Oxidation with H_2_O_2_ and KMnO_4_ on the Structure and Performance of Flexible Polyurethane/Ground Tire Rubber Composite Foams

**DOI:** 10.3390/ma14030499

**Published:** 2021-01-21

**Authors:** Aleksander Hejna, Adam Olszewski, Łukasz Zedler, Paulina Kosmela, Krzysztof Formela

**Affiliations:** Department of Polymer Technology, Gdańsk University of Technology, Gabriela Narutowicza 11/12, 80-233 Gdańsk, Poland; adam.olszewski1995@gmail.com (A.O.); lukasz.zedler@pg.edu.pl (Ł.Z.); paulina.kosmela@pg.edu.pl (P.K.); krzform1@pg.edu.pl (K.F.)

**Keywords:** polyurethane foam, ground tire rubber, rubber modification, compatibility, recycling

## Abstract

The use of waste tires is a very critical issue, considering their environmental and economic implications. One of the simplest and the least harmful methods is conversion of tires into ground tire rubber (GTR), which can be introduced into different polymer matrices as a filler. However, these applications often require proper modifications to provide compatibility with the polymer matrix. In this study, we examined the impact of GTR oxidation with hydrogen peroxide and potassium permanganate on the processing and properties of flexible polyurethane/GTR composite foams. Applied treatments caused oxidation and introduction of hydroxyl groups onto the surface of rubber particles, expressed by the broad range of their hydroxyl numbers. It resulted in noticeable differences in the processing of the polyurethane system and affected the structure of flexible composite foams. Treatment with H_2_O_2_ resulted in a 31% rise of apparent density, while the catalytic activity of potassium ions enhanced foaming of system decreased density by 25% and increased the open cell content. Better mechanical performance was noted for H_2_O_2_ modifications (even by 100% higher normalized compressive strength), because of the voids in cell walls and incompletely developed structure during polymerization, accelerated by KMnO_4_ treatment. This paper shows that modification of ground tire rubber is a very promising approach, and when properly performed may be applied to engineer the structure and performance of polyurethane composite foams.

## 1. Introduction

Polyurethane (PU) foams are a versatile group of materials commonly applied in various industry branches due to their very broad spectrum of potential properties [1]. Their structure and properties are directly influenced by their chemical composition and the ratio of the two most important components applied during polymerization—Polyols and isocyanates. In the simplest terms, polyurethane foams may be divided into rigid and flexible foams [2]. They are used in different applications, but both groups are prone to potential innovations. Just as in the case of other polymer materials, PU foams are often applied as matrices for polymer composites [3]. Like other materials, one of the main trends associated with foamed PU composites is the search for new fillers, preferably from renewable resources or byproducts of other processes and products [4,5,6]. Such a phenomenon is commonly observed and is driven by economic and ecological factors. The introduction of such materials could noticeably reduce the use of conventional, petroleum-based raw materials required to manufacture polyurethanes [7]. Among the potential filler candidates for polyurethane foams are the following: polyurethane foam scraps [8], waste lignocellulose fillers [9,10], textiles [11], eggshell waste [12] and rubber wastes [13]. The first solution is present on the market and used to manufacture the underlays for floors or carpet linings [14]. These materials are obtained by the re-foaming of the flexible polyurethane foam waste. Due to the cellular structure of matrix (new foam) and filler (foam scraps), they act as an excellent insulating material and are characterized by a thermal conductivity coefficient in the range of 0.036–0.041 W/(m/K), which is lower than conventional expanded polystyrene (~0.044 W/(m/K)) or mineral wool (~0.055 W/(m/K)) [15]. Moreover, they may also act as acoustic insulation with a sound reduction improvement of 19–44 dB, depending on the thickness.

The cellular structure of polyurethane foam scraps is a great advantage compared to the lignocellulose fillers, textiles or eggshell waste, because it is not affected by the filler particle size [9]. In case of solid fillers, their size and surface development noticeably affect the foaming of the polyurethane system, cellular structure and performance of resulting composites. Typical applications of PU foams such as damping, insulation or sound absorption require a well-developed cellular structure and are very sensitive towards changes [3]. Therefore, filler properties should be properly adjusted, which may require additional operations, especially considering that these materials themselves are rigid and do not show excellent damping or insulation performance. This could be the reason such materials are not industrially produced and applied as insulation materials.

The last material, rubber waste, is an auspicious one, due to the excellent mechanical properties of many primary rubber materials, e.g., car tires. They are commonly used in mechanical recycling resulting in the production of ground tire rubber (GTR). This material can be efficiently introduced into various polymer matrices, including polyurethane foams [16]. Contrary to the lignocellulose materials or textiles, GTR seems to be a more promising material due to its structure and properties. Waste rubber is a viscoelastic material, which when applied as a filler may enhance the damping [17] and sound absorption [18,19] performance of polymeric materials. Therefore, in foamed polyurethane composites it may act similarly to the waste polyurethane scraps, despite the lack of cellular structure.

The main factor limiting the application of ground tire rubber in polymer composites is the insufficient compatibility with polymer matrices. As a result, interfacial interactions between GTR applied as a filler and the continuous polymer phase is too weak for efficient stress transfer. The strength of materials is reduced, limiting one of rubber’s main advantages—an excellent mechanical performance. The proper adhesion between phases is particularly important in the case of foamed composites, e.g., based on polyurethanes (PU), whose mechanical performance is strictly associated with the apparent density, which is proportional to the share of solid material [20]. Therefore, it is essential to enhance the interfacial interactions between the matrix and filler. As mentioned above, polyurethanes are obtained by the reactions between polyols and isocyanates. Therefore, to enhance the compatibility of GTR with PU, it is beneficial to introduce hydroxyl or isocyanate functional groups onto its surface. The more feasible approach is related to the hydroxyl groups, which can be introduced during partial devulcanization or oxidation of the GTR surface [21]. Different approaches to GTR surface treatment resulting in its activation and incorporation of functional groups have been reported in the literature, which was aimed at the enhancement of adhesion with polymer matrices [22].

In the presented research work, we aimed to investigate GTR treatment’s impact with hydrogen peroxide and potassium permanganate on its structure and properties. We proposed the titration-based method to evaluate modification effectiveness for the potential applications of modified GTR in manufacturing polyurethane-based composites. Moreover, modified GTR samples were introduced into a flexible foamed PU matrix. The influence of GTR modifications on the processing of polyurethane systems (kinetic profile of foaming, processing times and temperatures), cellular structure (scanning electron microscopy, helium pycnometry), mechanical (static compression tests), and thermal (thermogravimetric analysis) properties were determined.

## 2. Materials and Methods

### 2.1. Materials

Ground tire rubber (GTR) obtained by ambient grinding of used tires (a combination of passenger car and truck tires in 50:50 mass ratio), whose average particle size is approximately 0.6 mm, was produced and provided by Recykl S.A. (Srem, Poland).

The 30% solution of hydrogen peroxide and crystals of potassium permanganate were acquired from Sigma Aldrich (Poznan, Poland). During the evaluation of the chemical structure of modified GTR the following chemicals were applied: acetone, dibutylamine, chlorobenzene, hydrochloric acid, technical grade toluene diisocyanate (TDI) and 3′,3″,5′,5″-tetrabromophenolsulfonphthalein. All chemicals were acquired from Sigma Aldrich (Poznan, Poland) and were used as received.

Polyurethanes were synthesized from a commercially available polyurethane system consisting of SPECFLEX^®^ NF 706 polyol and SPECFLEX^®^ NE 434 isocyanate, acquired from M. B. Market Ltd. (Czestochowa, Poland). The densities of used components at 25 °C were equal to 1.03 and 1.21 g/cm^3^, respectively, while their viscosity values at 25 °C equal 1340 and 66 mPa/s. According to the manufacturer, an applied polyurethane system is recommended to produce highly flexible, formed polyurethane foams.

### 2.2. Modifications of GTR

GTR was modified with a 30% solution of hydrogen peroxide and a 15% solution of potassium permanganate. The solution of potassium permanganate was prepared by dissolving its crystals in distilled water. Particles of ground tire rubber and the proper solution in different weight ratios: 1:2, 1:1 and 2:1, were mixed for 5 min at room temperature using a mechanical stirrer. They were left in solutions at room temperature for 72 h, then strained and dried at 70 °C for 8 h. For comparison, GTR dried at 70 °C for 8 h was used as reference.

### 2.3. Preparation of Polyurethane/GTR Composite Foams

Polyurethane/GTR composite foams were prepared on a laboratory scale by a single-step method. Predetermined amounts of polyol and isocyanate were mixed at a 100:70 mass ratio for 5 s at 1800 rpm. In the case of modified foams, incorporated fillers were previously mixed with polyol components for 1 min at 1800 rpm. Total mass of foam was set at 150 g. The resulting mixture was left for a free rise. After, the samples were conditioned at room temperature for 24 h. Table 1 contains the details of foam formulations.

### 2.4. Measurements

Changes in the chemical structure of GTR were evaluated using a modified method for the determination of free isocyanate group content by titration with dibutylamine, according to ASTM D-2572 [23]. The 0.5 g samples of GTR were put in a glass flask with 0.5 g of toluene diisocyanate and 20 cm^3^ of acetone. Mixtures were thoroughly mixed, sealed and stored at room temperature for 24 h. Then, proper amounts of dibutylamine solution in chlorobenzene and 3′,3″,5′,5″-tetrabromophenolsulfonphthalein were added. Then, mixtures were titrated with 0.1 M hydrochloric acid until the color changed to yellow. Obtained results were compared with the free isocyanate content of neat toluene diisocyanate to determine the number of functional groups at the rubber surface able to react with isocyanates. Such evaluation is essential for the potential application of modified GTR as a filler for polyurethane materials.

The free isocyanate content of the GTR/TDI mixture (%_NCO_) was calculated according to the following Equation (1):

%_NCO_ = (4.202 × (*V*_B_ − *V*_S_) × *N*_HCl_)/*m*_TDI_ (1)

where: *V*_B_—The volume of HCl required for titration of the blank sample, ml; *V*_S_—The volume of HCl required for titration of analyzed sample, ml; *N*_HCl_—Molarity of HCl, M; *m*_TDI_—The mass of TDI placed in the flask, g.

Based on these values, the assumed hydroxyl numbers (*L*_OH_) of GTR were calculated. During calculations, it was assumed that all of the consumed isocyanate groups reacted with the GTR particles. Another assumption was that all of the functional groups present on the surface of GTR were hydroxyls. Considering these assumptions, the number of hydroxyl groups, which took part in reactions was calculated following the Equation (2):

*X*_OH_ = *X*_NCO_ = ((%_NCO-TDI_ − %_NCO_) × *m*_TDI_ × 2)/(*M*_TDI_ × 100) (2)

where: %_NCO-TDI_—Free isocyanate content in TDI, equal to 42.7%; *M*_TDI_—The molar mass of TDI, equal to 174.2 g/mol.

Then, the hydroxyl number of GTR was calculated from the Formula (3):

*L*_OH_ = 56,100 × *X*_NCO_/*m*_GTR_ (3)

where: *m*_GTR_—The mass of GTR placed in the flask, g.

The thermogravimetric (TGA) analysis of GTR and composites was performed using the TG 209 F3 apparatus from Netzsch (Selb, Germany). Samples of composites weighing approx. 10 mg were placed in a ceramic dish. The study was conducted in an inert gas atmosphere—Nitrogen in the range from 30 to 900 °C with a temperature increase rate of 10 °C/min.

For all the samples, the following processing times were determined: rise time (time of volumetric expansion) and the tack-free time (from the end of volumetric expansion to the point when the surface stopped being tacky to the touch). Moreover, during polymerization, the temperature of the foam surface was measured with infrared thermal imaging camera model Testo 872 (Testo SE & Co. KGaA, Lenzkirch, Germany).

After conditioning, foamed polyurethane composites were cut into samples whose properties were later determined following the standard procedures.

The samples’ morphology was evaluated by using a Hitachi model S3400 (Tokyo, Japan) scanning electron microscopy.

The samples’ apparent density was calculated following PN-EN ISO 845 [24], as a ratio of the sample weight to the sample volume (g/cm^3^). The cube-shaped samples were measured with a slide caliper with an accuracy of 0.1 mm and weighed using an electronic analytical balance with an accuracy of 0.0001 g.

The content of open cells in foamed PU/GTR composites was determined using Ultrapyc 5000 Foam gas pycnometer from Anton Paar (Warszawa, Poland). The following measurement settings were applied: gas—Helium; target pressure—3.0 psi; foam mode—On; measurement type—Uncorrected; flow direction—Sample first; temperature control—On; target temperature—20.0 °C; flow mode—Monolith; cell size—Small, 10 cm^3^; preparation mode—Flow; time of the gas flow—0.5 min.

The compressive strength of studied samples was estimated following ISO 604 [25]. The cylindric samples with dimensions of 20 mm × 20 mm (height and diameter) were measured with a slide caliper with an accuracy of 0.1 mm. The compression test was performed on a Zwick/Roell Z020 tensile tester (Ulm, Germany) at a constant speed of 15%/min until reaching 70% deformation.

## 3. Results and Discussion

### 3.1. Changes in GTR Structure

**Figure 1:**
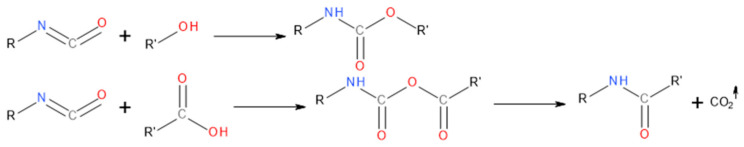
Possible reactions of isocyanates with functional groups present on the surface of modified GTR.

In Table 2, the free isocyanate contents (%_NCO_) of GTR/TDI mixtures prepared according to developed methodology and the decrease of their value comparing to neat TDI (Δ_NCO_) are shown. Before the modification, GTR particles also contained the functional groups able to react with isocyanates on its surface. Considering the data presented by Vilar [26], at room temperature and without the catalyst, isocyanates are reacting the most rapidly with amines (relative reaction rates from 200 for aromatic amines to even 100,000 for primary aliphatic ones), which are hardly present in GTR. Lower reaction rates are noted for primary hydroxyls and water (100), followed by carboxylic acids (40), secondary hydroxyls (30) and ureas (15) [26]. Therefore, the presence of these groups determines the reactivity of ground tire rubber particles with isocyanates. The potential reactions with the free isocyanate groups are presented in Figure 1.

The TDI’s initial free isocyanate content mixed with unmodified GTR equaled 33.3%, indicating its drop by 9.4%. As a result, the hydroxyl number of neat GTR was determined as 61.7 mg KOH/g. The presence of the functional groups on the surface of unmodified GTR was associated with the shredding of tires, which is performed under air atmosphere, which together with the high shear forces enables oxidation of rubber particles [27].

**Figure 2:**
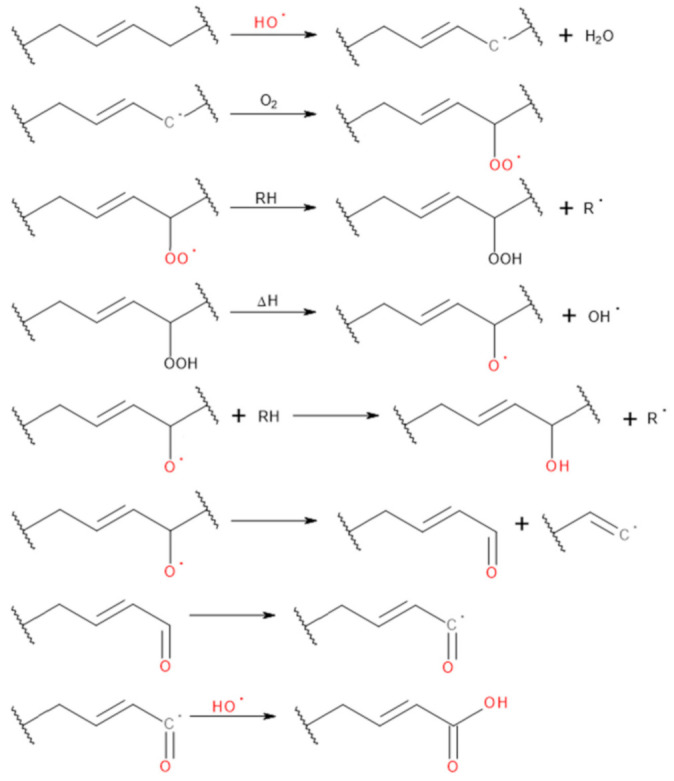
Schematic reactions occurring during oxidation of rubber.

Performed modifications of GTR caused noticeable changes in the chemical structure of their surface. It can be seen that the introduction of hydrogen peroxide resulted in the rise of the free isocyanate content, pointing to the reduced reactivity of GTR with TDI. Previous reports indicated rubber surface activation by creating carboxylic sites [28]. Hydrogen peroxide causes the generation of carbonium ions on the surface, which are converted into carboxylic sites. Potential reactions are presented in Figure 2. According to Shatanawi et al. [25], such an effect may be used to enhance the interactions between modified rubber and asphalts. A similar phenomenon was noted by Yehia et al. [29] for natural rubber vulcanizates. Moreover, they confirmed the generation of carboxyl groups by FTIR analysis.

In the presented case, the hydrogen peroxide treatment probably caused oxidation of the hydroxyls present on the surface of GTR particles, which resulted in the generation of carboxyl groups [30]. As mentioned above, carboxyls show lower reactivity with isocyanate groups. Hence, the hydroxyl number determined by the applied methodology was reduced. Although presented values should not be treated as the quantitative indicator or hydroxyl group content, they could be helpful for the adjustment of polyurethane foam recipes because they represent the content of groups reacting with isocyanate—One of the main components of polyurethanes.

On the other hand, potassium permanganate application caused a significant drop in GTR/TDI mixtures’ isocyanate content. Such an effect is associated with the chemical reactions occurring during the modification. As commonly known, KMnO_4_ is a strong oxidizing agent, independently of the media and pH value. In neutral solution, which was applied for modification it gets reduced to the brown manganese dioxide and four OH^−^ groups are released [31]. Moreover, when KMnO_4_ is attacking the alkene double bond, which could be present in GTR, two hydroxyl groups are generated [32]. As a result, modification with KMnO_4_ resulted in very high hydroxyl numbers. Therefore, compared to the H_2_O_2_, potassium permanganate could be considered a more effective activator of rubber particles’ surface aimed at the preparation of polyurethane materials.

Potassium permanganate is a highly reactive oxidizer. Even at ambient temperature, a violent reaction occurs with the release of MnO_2_ and molecular oxygen. The aforementioned compound application as a GTR surface modifier was studied before by Sonnier et al. [33], who confirmed the GTR surface oxidation phenomenon by a 2% solution of KMnO_4_ resulting in the creation of carbonyl groups. In the present study, GTR was treated with a 15% solution of the compound at three different ratios to conduct the process in a highly aggressive reaction environment. The treatment was also done using a 30% solution of H_2_O_2_, which also oxidizes the surface of GTR [29].

**Figure 3:**
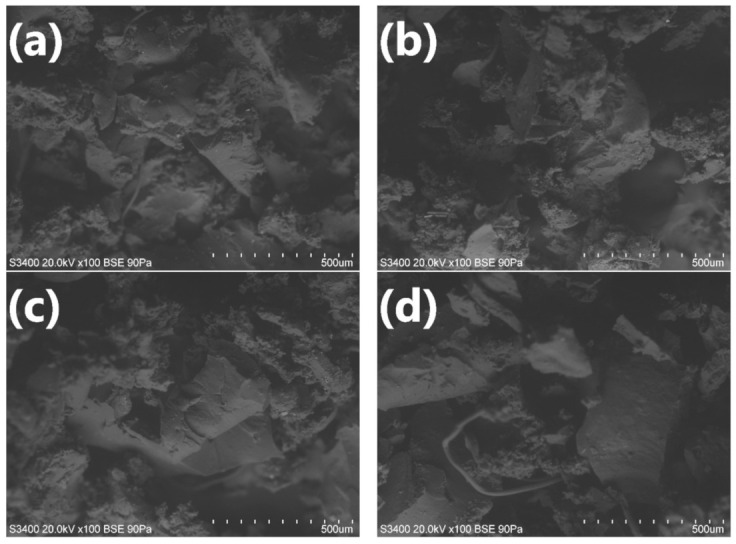

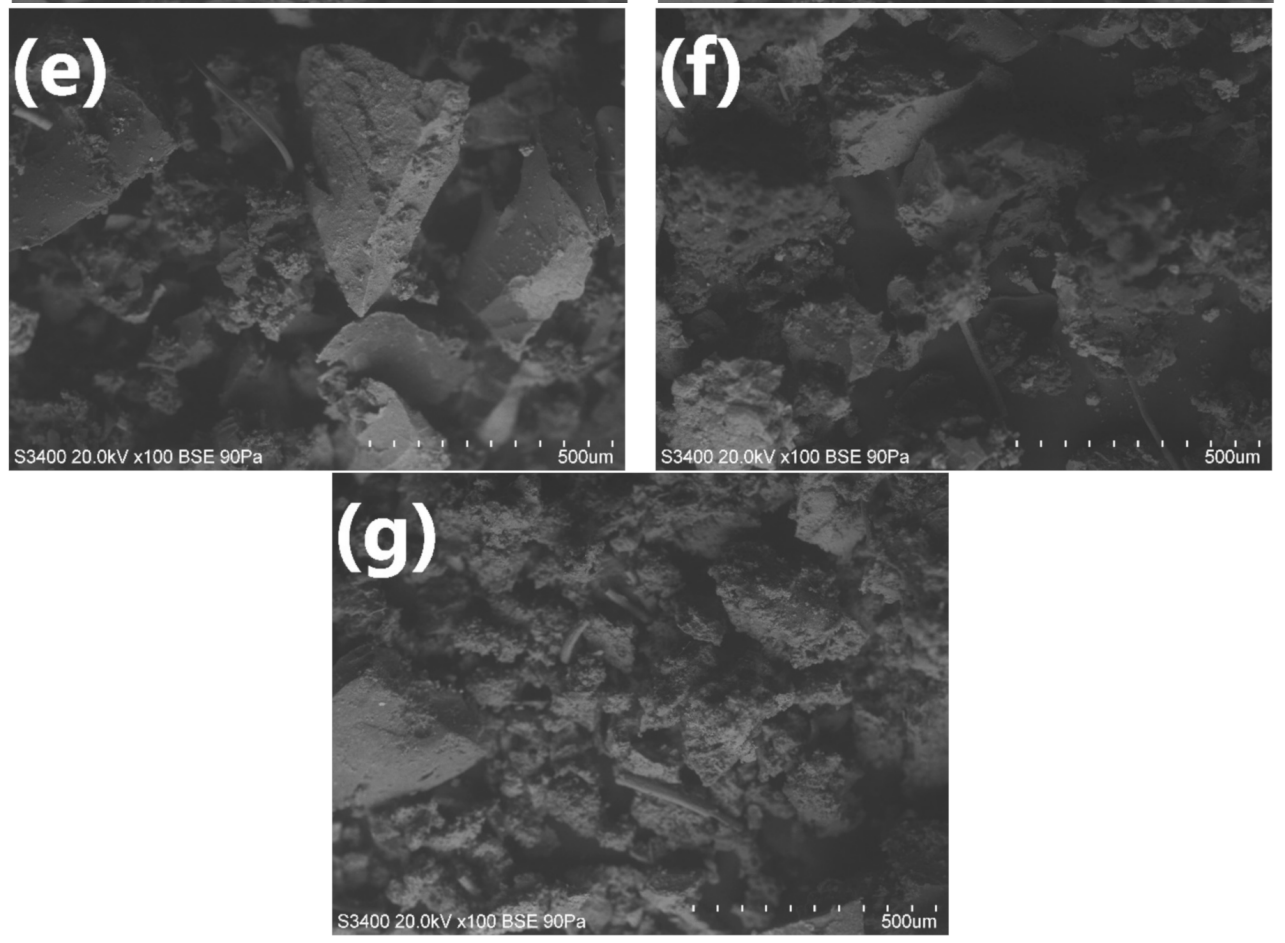
SEM images of studied samples: (**a**) neat GTR; (**b**) GTR:H_2_O_2_ 2:1; (**c**) GTR:H_2_O_2_ 1:1; (**d**) GTR:H_2_O_2_ 1:2; (**e**) GTR:KMnO_4_ 2:1; (**f**) GTR:KMnO_4_ 1:1 and (**g**) GTR:KMnO_4_ 1:2 (magnification ×100).

The impact of H_2_O_2_ and KMnO_4_ treatment on the morphology of GTR is presented in Figure 3. Compared to unmodified GTR, the surface of H_2_O_2_ is more developed [34], however, it does not change significantly with the higher content of the applied modifier. It is in line with hydroxyl numbers determined for GTR:H_2_O_2_ 2:1, GTR:H_2_O_2_ 1:1 and GTR:H_2_O_2_ 1:2. Increased roughness is also noticeable for KMnO_4_ modified samples and it is more developed with an increasing amount of the oxidizer. This phenomenon is correlated with the oxidation of the GTR surface and the formation of MnO_2,_ which was not removed at the end of the modification. The more of the substrate is used, the more products are being formed, influencing the morphology of GTR. More developed specific surface area and potentially formed groups obtained via H_2_O_2_ and KMnO_4_ oxidation may improve compatibility between PU matrix and GTR filler, thereby changing the mechanical properties of PU/GTR materials.

**Figure 4:**
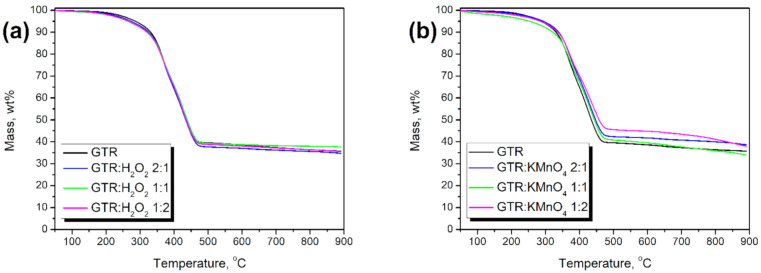
Results of thermogravimetric analysis of applied GTR fillers modified with (**a**) H_2_O_2_ and (**b**) KMnO_4_.

Thermogravimetric analysis (TGA) is a convenient method to analyze the changes in the chemical structure of reclaimed/modified rubber [35,36]. To evaluate the applied chemical treatment on GTR, the thermal stability, characteristic peaks and the amount of final residue of the neat and modified GTRs were measured and analyzed. The obtained results are presented in Table 3 and Figure 4.

Two characteristic peaks related to the two main components of GTR were noticed at approx. 362.8–373.6 °C (*T*_max1_) and at approx. 424.0–438.7 (*T*_max2_). These respectively correspond to thermal degradation of natural rubber and styrene-butadiene rubber [37], which proves that used material is taken from waste tires. All samples were characterized by *T*_max1_ and *T*_max2_ close to values for the reference sample, whereas the largest deviation from the neat GTR had sample GTR:KMnO_4_ 1:2 (*T*_max1_—6.1 °C and *T*_max2_—14.7 °C). While the deviation can be treated as a measurement error (~1.6 and 3.3%, respectively), it still varies significantly from the rest of the studied samples, especially the *T*_max2_ value. The shift of the temperature towards a higher value may be due to the start of thermal decomposition of MnO_2_ (483.0 °C) obtained during the oxidation reaction.

The difference in residue between the samples is negligible, although it is up to 7.5% by weight (GTR:KMnO_4_ 2:1) compared to the reference sample. This is because the analyzed sample is extremely small (approx. 10 mg), and GTR is a mix of car/truck tires that differs in composition. Those two factors influence the residue values.

The surface modifications change the thermal behavior of the studied samples. *T*_-2%_ values shift towards lower temperatures (215.4, 202.3, 204.1, 227.9, 213.0 and 204.0 °C for GTR:H_2_O_2_ 2:1, GTR: H_2_O_2_ 1:1, GTR: H_2_O_2_ 1:2, GTR:KMnO_4_ 2:1, GTR:KMnO_4_ 1:1 and GTR:KMnO_4_ 1:2, respectively) compared to the reference sample (233.0 °C for neat GTR), which resulted from the oxidation of the waste rubber. Moreover, as a result of the reactions, changes in the structure could occur and affect the volatilization of GTR unreacted components [38] and accelerate the process. The *T*_-5%_ values of KMnO_4_ modified GTR is similar to the reference sample (286.7, 293.6, 287.4 and 285.3 °C for neat GTR, GTR:KMnO_4_ 2:1, GTR:KMnO_4_ 1:1 and GTR:KMnO_4_ 1:2, respectively), while for GTR oxidized with H_2_O_2_ values drop (273.5, 266.9 and 265.1 °C for GTR:H_2_O_2_ 2:1, GTR: H_2_O_2_ 1:1 and GTR: H_2_O_2_ 1:2, respectively). H_2_O_2_, as well as KMnO_4_, oxidize the structure shifting thermal decomposition towards lower temperatures. However, due to the KMnO_4_ oxidation, MnO_2_ is being formed and not removed from the GTR. The presence of the component, which is characterized by high decomposition temperature (483.0 °C) influences the thermal stability as the temperature rises, which is also in accordance with *T*_-10%_ and *T*_-50%_ values. Significantly lower degradation temperatures of H_2_O_2_ modified materials may also indicate higher oxidation efficiency of GTR compared to KMnO_4_.

### 3.2. Kinetics of Foaming

**Figure 5:**
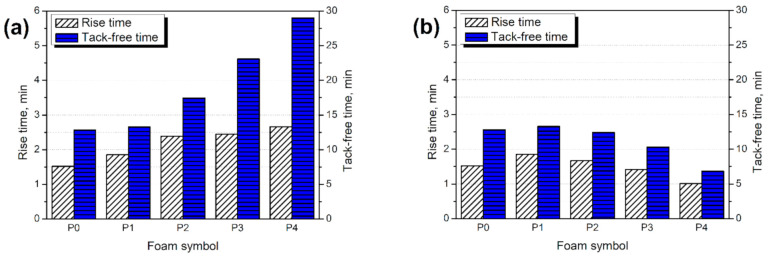
Processing times of reference foam, foam containing neat GTR (**a**) GTR modified with H_2_O_2_, and (**b**) GTR modified with KMnO_4_.

In Figure 5, there are presented values of rise time and tack-free time, depending on the foam formulation. The introduction of GTR into the polyol mixture resulted mainly in the elongation of rise time. Such an effect was related to increased viscosity of the polyol mixture due to the incorporation of solid rubber particles, which was also noted in our previous works [39,40,41]. Nevertheless, in these works, we investigated the rigid polyurethane foams, so different material behavior was noted. Higher isocyanate excess was applied, so the matrix was “stronger” during volumetric expansion and less sensitive to increased viscosity, even with isocyanate groups’ partial attraction by functional groups of GTR. For flexible foams, when isocyanate:hydroxyl ratio in the system is lower, the attraction of isocyanate groups by GTR causes the “weakening” of the polyurethane matrix, and hence the elongation of rise time.

Modification of GTR with hydrogen peroxide resulted in elongation of the processing times. Such an effect could be associated with the noticeable increase of the polyol mixture’s viscosity related to the incorporation of GTR. A similar phenomenon was noted in our previous works [39,40]. As shown in the SEM images of modified GTR (Figure 3), treatment with hydrogen peroxide caused surface development, which resulted in the enhanced interactions with the polyol mixture.

For modification with KMnO_4_, processing times were noticeably shortened, which was related to enhanced catalytic activity due to potassium permanganate’s basic character [26]. Basic catalysts are commonly applied in polyurethane synthesis and are not selective. They show strong catalytic activity in reactions with hydroxyl groups and water, but also with ureas and urethanes [26]. Therefore, they are accelerating all polyurethane foam synthesis steps, which can be seen in Figure 5, indicating a noticeable reduction of the rise and tack-free times. In the presented case, the catalytic effect was so strong that it overcame the viscosity increase associated with the significant development of the GTR surface after modification with potassium permanganate (see Figure 3).

**Figure 6:**
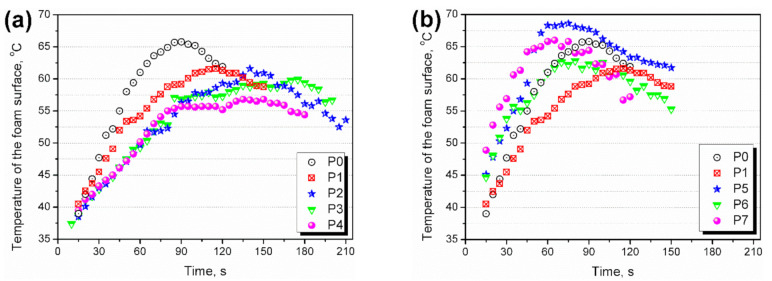
Temperature build-up on the surface of foams containing (**a**) GTR modified with H_2_O_2_ and (**b**) GTR modified with KMnO_4_.

**Figure 7:**
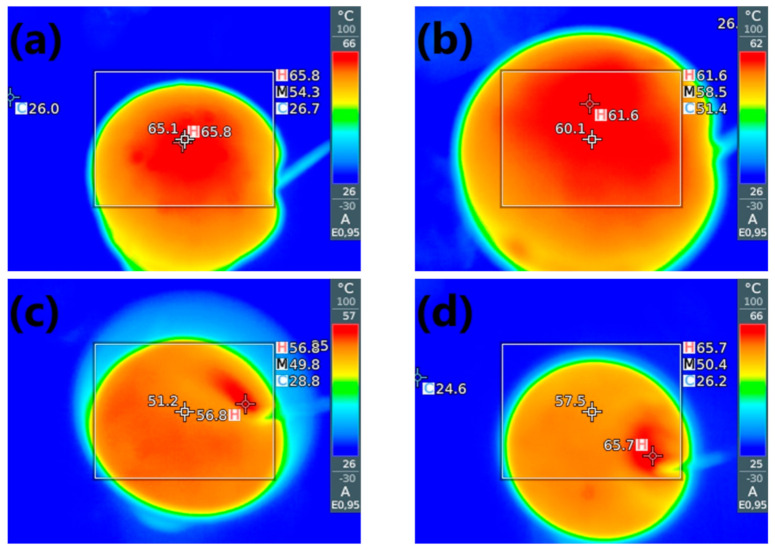
Photographs from thermographic camera showing the maximum temperatures during foaming of (**a**) P0, (**b**) P1, (**c**) P4, and (**d**) P7 samples.

In Figure 6, the presented graphs show the temperature build-up on the foam surface measured by the thermographic camera. Exemplary photographs are shown in Figure 7. The introduction of GTR into polyurethane foam reduced the maximum foam temperature during polymerization. It was associated with the interactions between GTR and isocyanates and confirms the results of previous works [39,40]. Moreover, the maximum temperature is reached almost 30 s later than for unfilled polyurethane foam, which confirms the processing times’ elongation due to the polyol mixture’s increased viscosity.

Modification of GTR by the hydrogen peroxide resulted in a slight reduction of the maximum foam surface temperature. It was caused by the increased viscosity of the polyol mixture related to the more developed surface area of modified rubber particles (see Figure 3). Due to the retardation of polymerization, heat build-up inside the foam was lower, and due to the heat convection to the environment, lower surface temperatures were noted.

In the case of KMnO_4_ modification, the catalytic activity fastened the temperature rise compared to P0 and P1 samples and increased the maximum temperature observed at the rising foam surface. Due to the acceleration of chemical reactions, the heat generated during polymerization could not dissipate and was accumulated inside the foam, increasing its temperature.

### 3.3. Structure and Properties of PU/GTR Composite Foams

**Figure 8:**
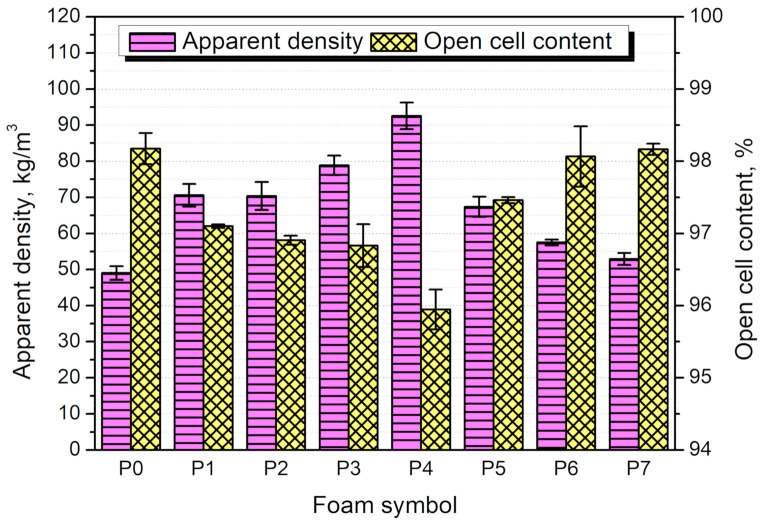
Values of the apparent density and open cell content for prepared PU/GTR composite foams.

In Figure 8, there are presented values of the apparent density and open cell content, parameters of foams’ cellular structure directly influenced by the course of the polymerization process. Incorporation of GTR into the foamed polyurethane matrix resulted in a noticeable increase in apparent density, which was associated with changes in the viscosity of the polyol mixture and differences in density between matrix and filler. The apparent density of foam P1 was noticeably affected when GTR particles applied as a filler were subjected to oxidation with hydrogen peroxide and potassium permanganate. However, significantly different behavior was noted for both modifiers.

The pretreatment of GTR particles with the hydrogen peroxide caused an increase in apparent density proportional to the applied modifier content. Such an effect is associated with the elongation of the processing times. As mentioned above, peroxide treatment of GTR resulted in the significant development of particles’ surface area, causing the enhanced viscosity of the reacting mixture and reducing the level of foams’ volumetric expansion. On the other hand, the catalytic effect of applied KMnO_4_ treatment resulted in the acceleration of all reactions occurring inside the foam, including the generation of carbon dioxide. Moreover, due to the higher temperature of the reacting mixture, its viscosity was lower, and gas could evaporate faster, increasing the volumetric expansion. Therefore, the apparent density was significantly reduced, and foam P7 almost reached the level of unfilled foam (52.9 and 49.0 kg/m^3^, respectively).

The content of open cells inside foam was also related to the type of applied filler. Its value equaled 98.2% for unfilled foam, typical for flexible, open-cell foams [42]. Viscosity changes and a higher density of GTR, which reduced the foam’s volumetric expansion, also caused a slight drop of open cell content to 97.1%. Similar to the apparent density, modification of rubber particles with H_2_O_2_ caused changes related to the enhanced level of interfacial interactions. Increased viscosity of the polyol mixture caused trapping of carbon dioxide generated during the foaming of material. The adverse effect was noted for potassium permanganate treatment. Due to accelerated foam rise and the lower level of the interactions between GTR and polyurethane matrix, generated gas could rapidly foam the reacting mixture, which was simultaneously solidifying, and then escape from foam. The higher temperature of foam compared to hydrogen peroxide modification also facilitated the gas movement.

**Figure 9:**
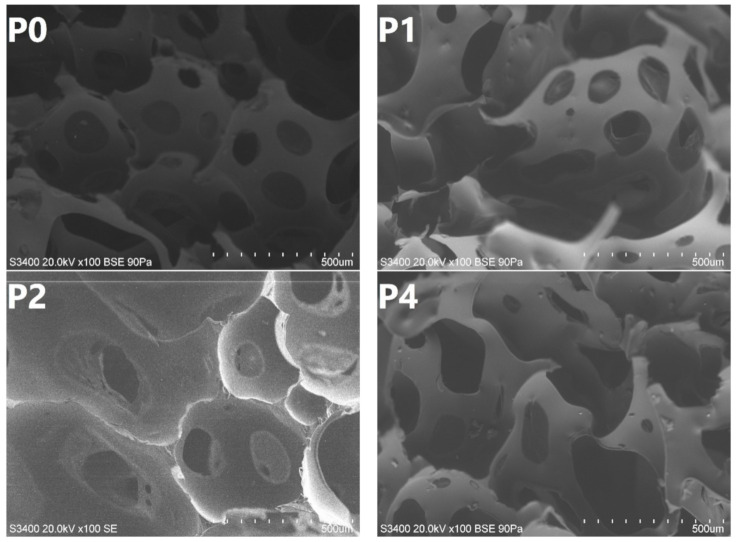

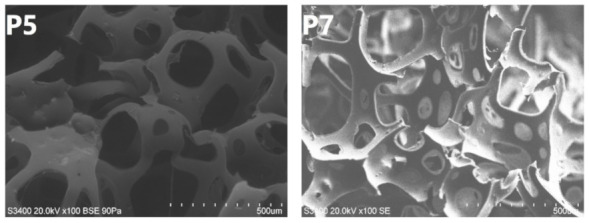
SEM images of prepared foams P0, P1, P2, P4, P5, and P7 (according to Table 1).

Figure 9 presents the images of foams’ cellular structure obtained with the scanning electron microscope. It can be seen that the reference foam, without rubber, shows relatively regular cells with noticeable holes in cell walls resulting in high open cell content. Such an effect is typical for the flexible polyurethane foams, which are often used in applications that require a high content of open cells [42]. The introduction of unmodified GTR caused some irregularities in the cell size and shape, which was also observed in our previous work and by other researchers [43,44]. Nevertheless, the disruption of the cellular structure was not very significant. For foams P2 and P4 containing the GTR modified with the hydrogen peroxide, the changes were more visible. A noticeable rise in cell size was observed, and for foam P4, the regularity of the structure was disrupted. It was probably associated with enhancing the polyol mixture’s viscosity caused by the higher specific weight of GTR and enhanced interactions with polyurethane components. In the SEM image of sample P4, it can be seen that the cellular structure was even collapsed, which contributed to the increase in the foam’s apparent density. For foams containing KMnO_4_ modified GTR, changes were also evident. Due to the catalytic activity of potassium ions, the cells were bigger and with a higher portion of holes. Such an effect was related to the accelerated gas evaporation during foaming and increased the open cell content in foams (see Figure 8).

**Figure 10:**
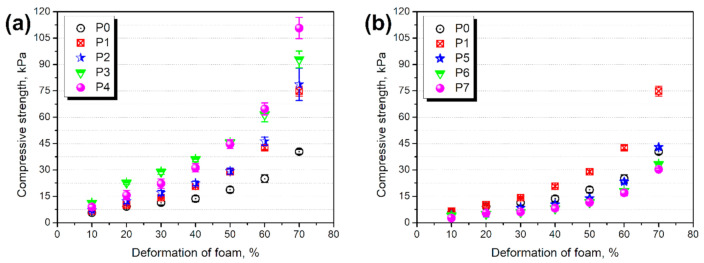
Compressive strength of foams containing (**a**) GTR modified with H_2_O_2_ and (**b**) GTR modified with KMnO_4_.

In Figure 10 there are presented values of the compressive strength of prepared polyurethane/ground tire rubber composites at various levels of deformation. It can be seen that the incorporation of GTR caused a noticeable increase in the foam’s strength. The compressive performance enhancement was even more substantial when GTR particles were pretreated with the hydrogen peroxide solution. Such an effect is associated with improved interfacial interactions related to rubber particles’ surface development (see Figure 3). On the other side, modification with KMnO_4_ caused a drastic drop of foams’ compressive strength, even below the values noted for neat, unfilled foam. Such an effect was related to the weakening of the PU cellular structure, caused by the significant increase of holes in cell walls (see Figure 9). As a result, the foams’ structure was able to withstand only low forces without breaking.

**Figure 11:**
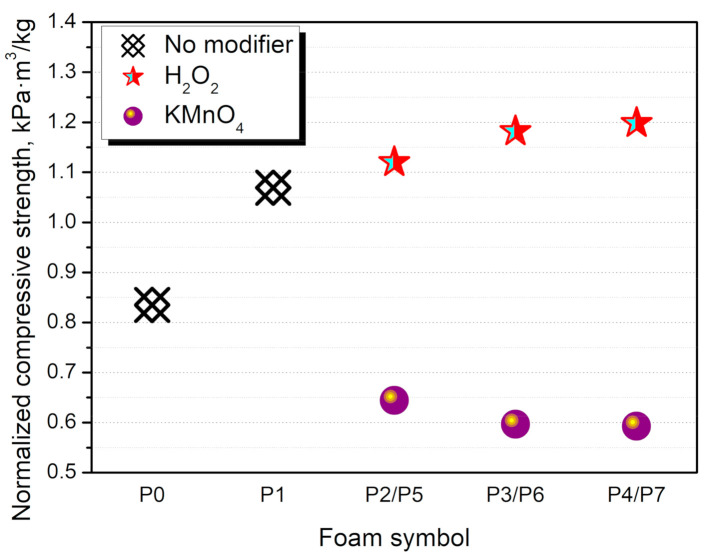
Normalized compressive strength of PU/GTR composite foams.

The compressive performance of cellular materials is strictly associated with their apparent density. A higher density of foam implicates a more compact structure, which can withstand higher forces. Therefore, for the real comparison of different cellular materials, the impact of apparent density should be eliminated. In Figure 11 there are presented normalized values of foams’ compressive strength. The incorporation of neat GTR results in a “true” reinforcement effect compared to the unfilled material. This effect was enhanced by the modification with H_2_O_2_ and its increased loading. For KMnO_4_, values are significantly lower, even despite the decreased apparent density, indicating that GTR did not cause a reinforcing effect after this modification. Such an effect could be associated with the changes in the isocyanate:hydroxyl ratio, due to the high hydroxyl numbers of KMnO_4_ modified GTR.

**Figure 12:**
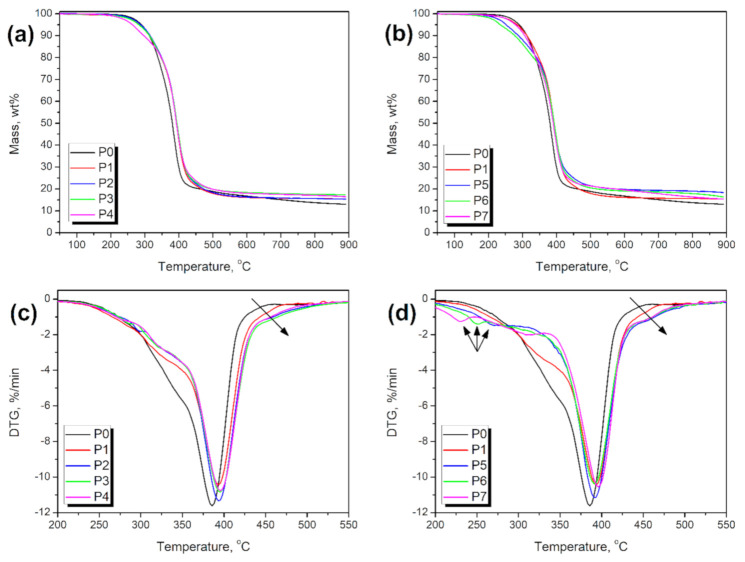
Plots of (**a**,**b**) mass loss and (**c**,**d**) differential thermogravimetric curves of foams containing (**a**,**c**) GTR modified with H_2_O_2_ and (**b**,**d**) GTR modified with KMnO_4_.

In Table 4, there are presented the results of the thermogravimetric analysis of prepared foams. Moreover, Figure 12 shows the course of degradation, and differential thermogravimetric curves, indicating the rate of decomposition at particular temperatures. They are presented only for the 200–550 °C temperature range, when the actual thermal degradation occurred. It can be seen that the reference foam is characterized by thermal stability typical for flexible polyurethane foams [45]. The onset of the thermal degradation process, measured as a temperature associated with the 2 wt.% mass loss, was determined as 264.2 °C. It is significantly above the temperature range required for the typical applications of the flexible polyurethane foams, which is usually in the range of 160–180 °C [26]. Reference foam is characterized by the almost single-step decomposition process, with the maximum rate of decomposition at 385.5 °C, which is typical for the polyurethane soft segment decomposition [46]. Some portion of the material may degrade at slightly lower temperatures, which corresponds to the decomposition of urethane bonds. The introduction of neat and modified GTR reduced the thermal stability of prepared foams. Such an effect may be related to the lower stability of introduced rubber particles than the polyurethane matrix, due to its oxidation (see Table 3). Similar effects were observed in our previous works, when partially devulcanized rubber was introduced into PU foams [39,40]. The effect was more pronounced for GTR modified with potassium permanganate, which is associated with the enhanced catalytic activity and weaker polyurethane matrix. Moreover, due to the high hydroxyl numbers of modified rubbers, the matrix could not be fully developed, and unbound macromolecular chains of polyol could reduce the decomposition temperature.

The introduction of rubber particles also resulted in changes in the course of decomposition, which was no longer a one-step process. The value of *T*_max3_, which is related to the main decomposition step, was shifted towards higher temperatures because of the presence of rubber and the excellent stability of the untreated, crosslinked parts, mostly styrene-butadiene rubber present in tires (see Table 3). It was marked with an arrow in Figure 10. Also, it can be seen that the values of *T*_max1_ and *T*_max2_ correspond to the decomposition of GTR—probably the oxidized part and the urethane and amide bonds generated during interactions of modified rubber with isocyanate groups. These bonds show lower thermal stability comparing to the soft segments originated from polyols’ macromolecules [46]. Also, for KMnO_4_ treatment, the incomplete development of matrix and unbound fragments of polyol chains could reduce material stability. For samples P5–P7, the additional degradation steps at lower temperatures are particularly noticeable.

## 4. Conclusions

The presented research paper aimed to investigate the impact of GTR treatment with hydrogen peroxide and potassium permanganate on its microstructure, chemical structure of surface and thermal stability. Applied modifications resulted in the development of GTR surface due to partial oxidation, which could be observed in the scanning electron microscope images. Moreover, application of KMnO_4_ significantly increased the hydroxyl number of modified GTR samples, associated with the incorporation of hydroxyl groups onto the surface. Partial oxidation of the surface of GTR slightly reduced the thermal stability of modified GTR samples. Nevertheless, the onset of degradation still exceeded the value of 200 °C, which guarantees the safe processing window for manufacturing of foamed polyurethane/GTR composites, without the further decomposition of rubber particles.

Modified GTR samples were introduced into a flexible foamed polyurethane matrix. The impact on the processing, cellular structure, mechanical and thermal performance was investigated. The introduction of neat GTR and GTR modified with hydrogen peroxide caused the elongation of processing times and reduced processing temperatures. Such an effect was mostly due to the increased viscosity of the polyol mixture, caused by the introduction of solid particles. The treatment with KMnO_4_ caused the opposite effect, due to the catalytic activity of potassium ions. Changes in processing were mirrored in the cellular structure of foams. For neat and H_2_O_2_-modified GTR, the typical rise of the apparent density and drop of open cell content was observed. At the same time, KMnO_4_ treatment catalyzed the polymerization, resulting in enhanced gas generation, reduction of the apparent density and increase of open cell content. The compressive properties were directly affected by the quality of cellular structure and apparent density of foams. Therefore, superior mechanical performance was observed for H_2_O_2_ modifications, because of the incompletely developed structure during polymerization accelerated by KMnO_4_ treatment. Considering thermal properties, changes in the chemical structure of modified GTR were reflected in changes of foamed polyurethane/GTR composites. Generally, the presented results indicate that the chemical modification of ground tire rubber should be considered an auspicious method for compatibilization of polyurethane/GTR composites. Through proper treatment of GTR, composites with the desired properties could be obtained. Future trends in this area should include modifications of GTR using continuous methods, enhancing the economic and ecological aspects of the process, as well as more in-depth evaluation of the changes during processing of polyurethane systems.

## Figures and Tables

**Table 1:** Formulations of prepared composite foams.

Component	Foam Symbol							
	P0	P1	P2	P3	P4	P5	P6	P7
	Component Content, wt.%							
Polyol	58	47	47	47	47	47	47	47
Isocyanate	42	33	33	33	33	33	33	33
GTR	-	20	-	-	-	-	-	-
GTR:H_2_O_2_ 2:1	-	-	20	-	-	-	-	-
GTR:H_2_O_2_ 1:1	-	-	-	20	-	-	-	-
GTR:H_2_O_2_ 1:2	-	-	-	-	20	-	-	-
GTR:KMnO_4_ 2:1	-	-	-	-	-	20	-	-
GTR:KMnO_4_ 1:1	-	-	-	-	-	-	20	-
GTR:KMnO_4_ 1:2	-	-	-	-	-	-	-	20

**Table 2:** Free isocyanate contents of GTR/TDI mixtures, amount of isocyanate groups of TDI consumed by GTR and calculated hydroxyl numbers of GTR.

Sample	%_NCO_, %	Δ_NCO_, %	*L*_OH_ mg KOH/g
GTR	33.3 ± 1.1	9.4 ± 1.1	61.7 ± 3.0
GTR:H_2_O_2_ 2:1	37.0 ± 0.3	5.7 ± 0.3	36.4 ± 1.6
GTR:H_2_O_2_ 1:1	37.3 ± 1.1	5.4 ± 1.1	34.5 ± 2.4
GTR:H_2_O_2_ 1:2	37.9 ± 1.1	4.8 ± 1.1	32.1 ± 2.3
GTR:KMnO_4_ 2:1	12.1 ± 0.5	30.6 ± 0.5	205.9 ± 9.9
GTR:KMnO_4_ 1:1	7.4 ± 0.5	35.3 ± 0.5	226.3 ± 6.2
GTR:KMnO_4_ 1:2	3.7 ± 0.2	39.0 ± 0.2	248.9 ± 3.3

**Table 3:** The results of thermogravimetric analysis of neat and modified GTR.

GTR Type	*T*_-2%_, °C	*T*_-5%_, °C	*T*_-10%_, °C	*T*_-50%_, °C	Residue_890 °C_, %	*T*_max1_, °C	*T*_max2_, °C
GTR	233.0	286.7	331.1	435.0	35.86	369.0	424.0
GTR:H_2_O_2_ 2:1	215.4	273.5	325.3	434.3	34.68	369.3	426.1
GTR: H_2_O_2_ 1:1	202.3	266.9	321.7	438.7	37.57	368.8	429.6
GTR: H_2_O_2_ 1:2	204.1	265.1	320.7	435.9	35.39	370.8	425.6
GTR:KMnO_4_ 2:1	227.9	293.6	338.9	447.3	38.56	369.5	432.0
GTR:KMnO_4_ 1:1	213.0	287.4	337.2	449.0	37.88	373.6	429.1
GTR:KMnO_4_ 1:2	204.0	285.3	336.6	457.8	37.85	369.2	438.7

**Table 4:** Results of thermogravimetric analysis for prepared foams.

Foam Symbol	*T*_-2%_, °C	*T*_-5%_, °C	*T*_-10%_, °C	*T*_-50%_, °C	Residue_890 °C_, %	*T*_max1_, °C	*T*_max2_, °C	*T*_max3_, °C
P0	264.2	290.1	313.0	380.7	12.98	-	-	385.5
P1	247.6	279.0	306.6	390.3	15.32	-	324.2	393.9
P2	249.2	284.2	313.2	392.9	15.33	297.8	-	393.8
P3	257.8	287.6	315.6	394.5	15.29	287.7	318.9	395.0
P4	248.7	282.2	313.6	394.6	17.24	278.7	322.1	395.7
P5	229.8	263.5	297.3	393.0	16.42	269.2	-	391.8
P6	221.8	251.9	287.9	392.3	18.32	251.5	-	391.7
P7	203.9	234.6	277.3	393.4	16.34	230.0	309.6	395.8

## Data Availability

Data is contained within the article. The data presented in this study are available in The Impact of Ground Tire Rubber Oxidation with H_2_O_2_ and KMnO_4_ on the Structure and Performance of Flexible Polyurethane/Ground Tire Rubber Composite Foams.
